# SILAC-Based Mass Spectrometry Analysis Reveals That Epibrassinolide Induces Apoptosis via Activating Endoplasmic Reticulum Stress in Prostate Cancer Cells

**DOI:** 10.1371/journal.pone.0135788

**Published:** 2015-09-09

**Authors:** Pinar Obakan, Carlos Barrero, Ajda Coker-Gurkan, Elif Damla Arisan, Salim Merali, Narcin Palavan-Unsal

**Affiliations:** 1 Istanbul Kultur University, Department of Molecular Biology and Genetics, Atakoy Campus, Bakirkoy, Istanbul-Turkey; 2 Department of Biochemistry, Temple University School of Medicine, Fels Institute, 3307 N. Broad Street, Philadelphia, Pennsylvania, United States of America; Hormel Institute, University of Minnesota, UNITED STATES

## Abstract

Epibrassinolide (EBR) is a polyhydroxylated sterol derivative and biologically active compound of the brassinosteroids. In addition to well-described roles in plant growth, EBR induces apoptosis in the LNCaP prostate cancer cells expressing functional androgen receptor (AR). Therefore, it is suggested that EBR might have an inhibitory potential on androgen receptor signaling pathway. However, the mechanism by which EBR exerts its effects on LNCaP is poorly understood. To address this gap in knowledge, we used an unbiased global proteomics approach, i.e., stable-isotope labeling by amino acids in cell culture (SILAC). In total, 964 unique proteins were identified, 160 of which were differentially expressed after 12 h of EBR treatment. The quantification of the differentially expressed proteins revealed that the expression of the unfolded protein response (UPR) chaperone protein, calreticulin (CALR), was dramatically downregulated. The decrease in CALR expression was also validated by immunoblotting. Because our data revealed the involvement of the UPR in response to EBR exposure, we evaluated the expression of the other UPR proteins. We demonstrated that EBR treatment downregulated calnexin and upregulated BiP and IRE1α expression levels and induced CHOP translocation from the cytoplasm to nucleus. The translocation of CHOP was associated with caspase-9 and caspase-3 activation after a 12 h EBR treatment. Co-treatment of EBR with rapamycin, an upstream mTOR pathway inhibitor, prevented EBR-induced cell viability loss and PARP cleavage in LNCaP prostate cancer cells, suggesting that EBR could induce ER stress in these cells. In addition, we observed similar results in DU145 cells with nonfunctional androgen receptor. When proteasomal degradation of proteins was blocked by MG132 co-treatment, EBR treatment further induced PARP cleavage relative to drug treatment alone. EBR also induced Ca^2+^ sequestration, which confirmed the alteration of the ER pathway due to drug treatment. Therefore, we suggest that EBR promotes ER stress and induces apoptosis.

## Introduction

Brassinosteroids (BRs) are steroid-derived molecules with numerous physiological effects, including the regulation of hormonal balance, the activation of protein and nucleic acid synthesis, enzymatic activity, the cell cycle and cell growth [1, 2]. Beside the well-described effects in plants, their roles in mammalian cells are poorly understood and currently being investigated as anti-cancer agents [3–5]. The recent understanding is that EBR, a member of the BRs, induces apoptosis more effectively in nuclear hormone receptor (NHR)-expressing cancer cell lines, such as LNCaP prostate [with androgen receptor (AR)] [4] or MCF-7 breast cancer cell lines [with estrogen receptor (ER)] [3]. The structural similarity of EBR with mammalian steroids [6] has been suggested as possible reason for the hormonal specificity. However, the molecular basis of the EBR specificity has not been elucidated. Our previous experience indicated that although EBR (25 μM) was a strong apoptotic inducer in LNCaP (AR+) prostate cancer cells, it was also surprisingly effective in inducing apoptosis in DU 145 (AR-) cells. Importantly, EBR treatment was not cytotoxic for PNT1a normal prostate epithelial cells [4]. To better clarify the therapeutic potential of EBR, we investigated the whole proteome of LNCaP cells with or without EBR treatment.

The use of quantitative proteomic approaches is likely to provide information on the key molecular signatures and the detailed understanding of the involved targets [7]. SILAC (Stable Isotope Labeling by Amino Acids in Cell Culture) analysis is a mass spectroscopy (MS)-combined proteomic approach without radioactive labeling. SILAC relies on the incorporation of a given 'light' (^12^C labeled L-lysine or L-arginine) or 'heavy' (^13^C labeled L-lysine or L-arginine) form of the amino acid into the proteins. After a number of cell divisions, each particular amino acid is replaced by its isotope analog and incorporated into newly synthesized proteins [8]. In this study, we used the SILAC approach to explore the novel apoptotic potential of EBR in androgen responsive LNCaP prostate cancer cells.

We observed that EBR significantly affected the expression profile of 160 proteins involving in different cellular functions (cell cytoskeleton, nucleic acid and energy metabolism, cell death and protein ubiquitination) compared with untreated control samples. Endoplasmic reticulum (ER) resident calreticulin (CALR), a chaperone protein, was significantly downregulated among those 160 proteins. We determined that the levels of ER stress proteins were altered after EBR treatment in LNCaP AR (+), and the same profile was also observed in the non-functional AR-expressing DU145 prostate cancer cell line. Alterations in the ER stress biomarkers triggered apoptosis in each cell line; in LNCaP cells, apoptosis was induced by CHOP transactivation and translocation to the nucleus. The addition of rapamycin, as a translational repressor of mTOR (mammalian target of rapamycin), or MG132, a proteasome inhibitor that reduces the degradation of ubiquitin-conjugated proteins, altered EBR-induced apoptosis, suggesting that ER stress was activated following EBR treatment in LNCaP cells. To prove the relationship between ER mediated cell death mechanism after EBR treatment, CALR plasmid transfection was performed. EBR-induced cell viability loss was prevented in CALR+ LNCaP cells. In addition, when CALR+ LNCaP cells were treated with EBR, we did not observe ER stress mediated apoptotic induction, suggesting that CALR is an important target of EBR.

## Materials and Methods

### Chemicals and antibodies

Heavy lysine and arginine [(13C6, 15N2)-L-lysine and (13C6)-L-arginine] were obtained from Cambridge Isotope (Andover, MA); light amino acids (L-lysine and L-arginine) were obtained from Sigma (St, Louis, MO). The protease inhibitor cocktail was obtained from Sigma (St, Louis, MO). The antibodies against ER stress (BiP, CALNX, CALR, CHOP, IRE1α, PERK, ATF4, ATF6 and PDI) and apoptosis (caspase-12, caspase-3, caspase-9 and PARP) were obtained from Cell Signaling Technology (Danvers, MA, USA). Tunicamycin was purchased from Sigma (St, Louis, MO) and dissolved in DMSO (10 mg/ml). The pmCherry-tagged CHOP plasmid was purchased from Addgene (CHOP promoter (-649/+136) pmCherry-1, 36035). Calcium green dye to determine the intracellular Ca^2+^ levels was purchased from Molecular Probes (Eugene, OR, USA). Rapamycin and MG132 were purchased from Tocris (Ellisville, MI, USA) and Sigma (St, Louis, MO), respectively.

### Cell culture

LNCaP (CRL-1740) and DU 145 (HTB-81) human prostate cancer cell lines were obtained from American Type Culture Collection (ATCC). HEK-293 (CRL-1573) cells were used for the wild type CALR mRNA isolation and obtained from ATCC. Cells were grown in DMEM medium (Gibco; Invitrogen, Carlsbad, CA, USA), supplemented with 10% fetal bovine serum (PAN Biotech, Aidenbach, Germany) and 10,000 U penicillin/ml and 10 mg streptomycin/ml (PAN Biotech, Aidenbach, Germany). Cells were cultured at 37°C in a humidified 5% CO_2_ incubator (HERAcell 150; Thermo Electron Corporation, Waltham, MA, USA).

### Cell culture in SILAC media

SILAC DMEM (Pierce Biotechnology) was supplemented with 10% dialyzed fetal bovine serum (Thermo Scientific, Waltham, MA, USA), 1% streptomycin/penicillin. The heavy medium was supplemented with ^13^C_6_ L-arginine and ^13^C_6_, ^15^N_2_-L-lysine. The light medium was supplemented with normal L-arginine and L-lysine. For SILAC experiments, LNCaP cells were grown in parallel in either light or heavy media for 5 days, with media replacement every 24 h.

### 1-D SDS-PAGE separation and in-gel trypsin digestion

Total cell protein was isolated from LNCaP cells using RIPA buffer (25 mM Tris-HCl pH 7.6, 150 mM NaCl, 1% NP-40, 1% sodium deoxycholate, 0.1% SDS). Protein quantification was performed according to the Bradford method (Bio-Rad Protein Assay) [9]. Samples containing a combined 40 μg of total protein (20 μg ‘‘heavy” and 20 μg ‘‘light”) were diluted with Laemmli sample buffer (Bio-Rad, Hercules, CA, USA) containing 5% β-mercaptoethanol. The mixture were then heated for 5 min at 90°C and loaded onto 10% polyacrylamide gels.1-D SDS-PAGE separation was performed with mini Protean II system (Bio-Rad) at 200 V for 45 min. Bands were visualized with Simply Blue Safe Stain (Life Technologies, CA, USA), and lanes were sliced into 12 sections, which were diced into ~ 1x1 mm squares. After distaining with 50% v/v acetonitrile (ACN) in 25 mM ammonium bicarbonate buffer (bicarbonate buffer), proteins in gel pieces were reduced with 10 mM dithiothreitol (DTT) in bicarbonate buffer and alkylated by incubation with 50 mM iodoacetamide in bicarbonate buffer. After gel dehydration with 100% ACN, gel pieces were covered with approximately 50 μl of 12.5 μg/ml trypsin in bicarbonate buffer for in-gel digestion. Incubation for digestion was performed at 37°C for 12 h. Trypsin was inactivated with formic acid at 2% final volume, and peptides were extracted and cleaned using a C18 Tip column (ZipTips, Millipore, Medford, MA, USA), as previously described [10].

### GeLC-MS/MS and data analysis

Peptides were dried in a vacuum centrifuge and resuspended in 20 μL of 0.1% v/v trifluoroacetic acid (TFA)/H_2_O. Peptide samples were loaded onto 2-μg capacity peptide traps (CapTrap; Michrom Bio-resources) and separated using a C18 capillary column (15 cm 75 mm, Agilent) with an Agilent 1100 LC pump delivering mobile phase at 300 nl/min. Gradient elution using mobile phases A (1% ACN/0.1% formic acid, balance H_2_O) and B (80% ACN/0.1% formic acid, balance H_2_O) was as follows (percentages for B, balance A): linear from 0 to 15% at 10 min, linear to 60% at 60 min, linear to 100% at 65 min. The nano ESI MS/MS was performed using an HCT Ultra ion trap mass spectrometer (Bruker). ESI was delivered using a distal-coating spray Silica tip (id 20 μm, tip inner id 10 μm, New Objective, Ringoes, NJ). Mass spectra were acquired in positive ion mode, capillary voltage at -1100 V and active ion charge control trap scanning from 300 to 1500 m/z; using an automatic switching between MS and MS/MS modes, MS/MS fragmentation was performed on the two most abundant ions on each spectrum using collision-induced dissociation with active exclusion (excluded after two spectra and released after 2 min). The complete system was fully controlled by HyStar 3.2 software.

Mass spectra data processing was performed using Mascot Distiller (Version 2.4.3.3) with search and quantitation toolbox options. The generated de-isotoped peak list was submitted to an in-house Mascot server 2.4.0 for searching against the SwissProt database version 2013_01 (538849 sequences; 191337357 residues). Mascot search parameters were set as follows: species, Homo sapiens (20,233 sequences); enzyme, trypsin with maximal 2 missed cleavage; fixed modification: cysteine carbamidomethylation; variable modifications: methionine oxidation, Gln->pyro-Glu (N-term Q), Glu->pyro-Glu (N-term E), Label: 13C(6)15N(2) (K), and Label:13C(6) (R); 0.90-Da mass tolerance for precursor peptide ions; and 0.6 Da for MS/MS fragment ions. SILAC quantitation was performed in Mascot Distiller using SILAC K+8 R+6 quantitation method; SILAC ratios for heavy and light peptide pairs were calculated using the Simpsons integration method, minimum 1 peptide with unique sequence and 0.05 of significant threshold. The following criteria were used to evaluate protein identification: one or more unique peptides with ion score ≥45 and two or more unique peptides with ion score ≥30 (p<0.05 threshold); proteins identified were extracted using MS Data Miner (MDM) [11]. Quantified proteins with ≥ 2 and ≤ 0.5-fold change were selected and clustered by biological functions, pathway and network analysis using Ingenuity Pathway Analysis (IPA) software (www.ingenuity.com) for bioinformatics analysis.

### Construction of p-EGFP CALR plasmid

Total RNA was isolated from HEK-293 human embryonic kidney cells using TRIPure (Roche) according to the manufacturer’s indications. First strand cDNA was transcribed using iScript cDNA Synthesis Kit (Bio-Rad). CALR cDNA was amplified using designed CALR gene-specific cloning primers: 5’-CGGAGTCAACGGATTTGGTCGTAT-3’; reverse, 5’-GCCTTCTCCATGGTGGTGAAGAC-3’. The protocol included an initial denaturation step at 94°C for 3 min, followed by 30 cycles with 30 sec. of denaturation at 94°C, 30 sec of annealing at 55°C and 45 sec. of elongation at 72°C, followed by a final elongation step 72°C for 10 min. Ten microliters of PCR product and 1 μg pEGF-LC3.1 plasmids were digested with 10 U/μl EcoRI and BglII (Fermentas, St. Leon-Rot, Germany) for 30 minutes at 37°C. The digestion products were subjected to 1.5% agarose gel electrophoresis and then extracted from the agarose gel. Ligation of the PCR product:plasmid was produced using 1:1, 1:3 and 1:5 ratios with T4 DNA ligase (Fermentas, St. Leon-Rot, Germany). Recombinant DNA was transformed into HB101 cells that were prepared using the CaCl_2_ method. Positive clones were selected by using ampicillin (Amp) LB agar plates. All of the colonies were picked and grown in 1 ml of Amp+LB and incubated overnight at 37°C. Plasmids from every colony were isolated by using QIAPrep Spin Miniprep columns (Qiagen, Valencia, CA, USA). Plasmids from selected ten colonies were used as templates to amplify the CALR gene using cloning primers in PCR. One selected amplified positive plasmid was sequenced to confirm the Open Reading Frame (ORF) of the EGF-CALR fusion gene (İontek, Turkey). The sequenced recombinant plasmid was used for the overexpression experiments.

### Transient transfection of the pEGFP-CALR plasmid

LNCaP cells were seeded overnight on 6-well plates at a density of 3x10^5^ cells/well. Approximately 1 μg/μl pCMV-CALR in the presence of transfection reagent (Fugene HD, Roche, Mannheim, Germany) was prepared in serum-free media. The mixture was incubated for 15 minutes at room temperature and gently added dropwise onto cells. After 48-h plasmid transfection, cells were treated with 25 μM EBR, and total RNA was isolated for CALR expression analysis or protein extraction for western blotting.

### Transient transfection of CHOP promoter (-649/+136) pmCherry-1 plasmid

To determine the activity of CHOP, LNCaP cells were transfected with the reporter construct CHOP promoter (-649/+136) pmCherry-1 (Addgene plasmid 36035) using Fugene HD according to the manufacturer's instructions (Roche, Mannheim, Germany), and clones resistant to kanamycin (500 μg/ml Sigma, St Louis, MO, USA) were generated.

### Evaluation of apoptotic cell death by Annexin V–FITC staining

LNCaP cells were seeded in 6 well-plates (3x10^5^ cells/well) and treated with 25 μM EBR for 24 h. Both floating and adherent cells were collected, resuspended in Annexin V binding buffer and incubated with Annexin V–FITC and PI following manufacturer instructions (BD Biosciences, Bedford, MA). One thousand events per sample were acquired on the Accuri C6 (BD Biosciences). Fluorescence emissions were collected through 530-nm and 570-nm band-pass filters for FITC and PI, respectively. Data are presented as dot plots (Annexin fluorescence on the x-axis; PI fluorescence on the y-axis). The numbers present in the four quadrants represent the percentage of viable (lower left), necrotic (upper left), early apoptotic (lower right), and late apoptotic (upper right) cells evaluated using BD Accuri C6 software (BD Biosciences).

### Western blot analysis

Cells were cultured in 60-mm Petri dishes in complete medium. The media were discarded, and the cells were washed with ice-cold 1x PBS and lysed with ProteoJET mammalian cell lysis buffer (Fermentas, St. Leon-Rot, Germany). Total protein levels were determined using the Bradford method (Bio-Rad, Hercules, CA, USA) [9]. Total cell lysates were separated by 12% SDS–PAGE gels and electrotransferred onto polyvinylidenedifluoride (PVDF) membranes (Roche, Mannheim, Germany) subjected to electrophoresis. Membranes were washed in tris-buffered saline with Tween-20 (TBS-T) [10 mM Tris-HCl (pH 8.3), 0.05% Tween-20] (Tween 20, Sigma Ultra, St. Louis, MO, USA). The membranes were blocked in 5% skim milk containing TBS-T milk overnight at 4°C. PVDF membranes were incubated with buffer containing 5% (v:v) skim milk solution with appropriate antibodies CALR, CALNX, BiP, IRE1α, CHOP, ATF4, ATF6, PERK, PDI, Caspase 12, cleaved caspase 9, caspase 3, PARP, cleaved PARP, β-actin (each diluted 1:1000) and incubated at 4°C overnight. Anti-rabbit secondary antibody was prepared 1:1000 dilution in 5% (v:v) skim milk solution (CST, Denvers, MA, USA). Membranes were rinsed with TBS-Tween 20 and incubated with HRP-conjugated secondary antibodies at 4°C overnight. Following the addition of enhanced chemiluminescence reagent, signals from the HRP-coupled antibodies were detected using ChemiDoc MP Imaging System (Bio-Rad Laboratories, Hercules, CA). All results were replicated at least three times and representative blots were given.

### Measurement of intracellular Ca^2+^ levels

LNCaP prostate cancer cells were treated with EBR for 12 h, and intracellular Ca^2+^ levels were determined by calcium green-1 (1 μmol/L, Molecular Probes, Eugene, OR, USA) staining for 30 minutes. Flow cytometric analysis of stained cells was performed with a flow cytometer Accuri C6 (BD Biosciences, San Jose, CA, USA). Calcium green-1-stained cells were observed by fluorescence microscopy (excitation 506 nm, emission: 531 nm).

### Statistical analysis

Statistical significance was assessed using the one-tailed unpaired t-test. P<0.05 was taken as a level of significance. Western blot results were repeated at least three times. Band intensities were quantified using ImageJ software and normalized to β-actin.

## Results

### Proteome of EBR-treated LNCaP cells

To obtain a global perspective of changes in the entire proteome, LNCaP cells were treated with EBR (25 **μ**M) and subjected to SILAC treatment. In total, 964 unique proteins were identified by this technique. Changes greater than 2-fold (≥ 2 and ≤ 0.5) between light and heavy proteins were considered significant according to the cutoffs of previous studies [10]. Quantitative analysis between paired samples revealed that among the 964 proteins, 160 were significantly changed after 12 h EBR treatment (S1 Table).

### Functional characterization of identified proteins and bioinformatics analysis

**Fig 1:**
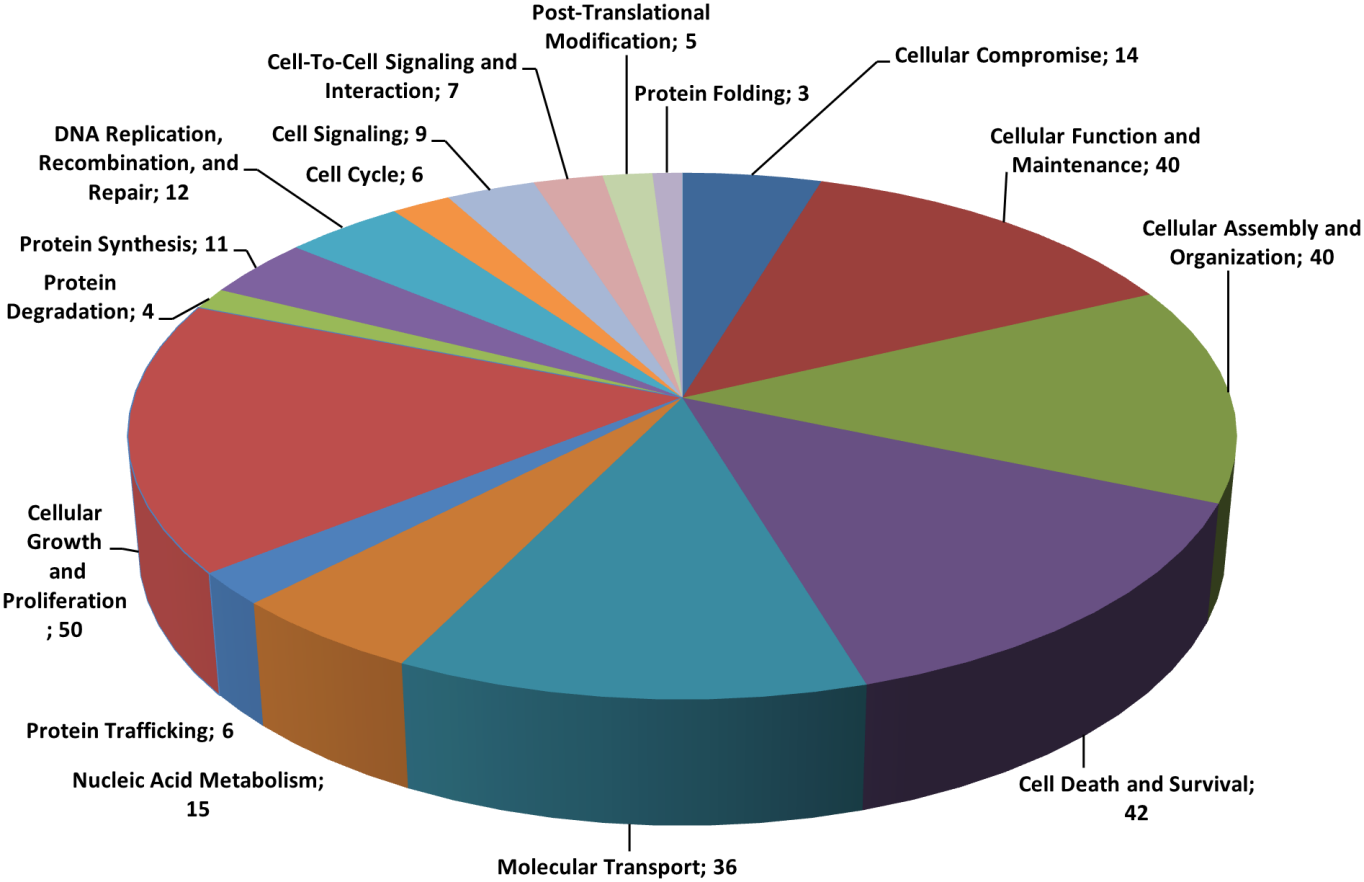
Categorization of the molecular function of differentially expressed proteins in EBR-treated LNCaP cells. The pie graph presents the 160 differentially expressed proteins.

**Fig 2:**
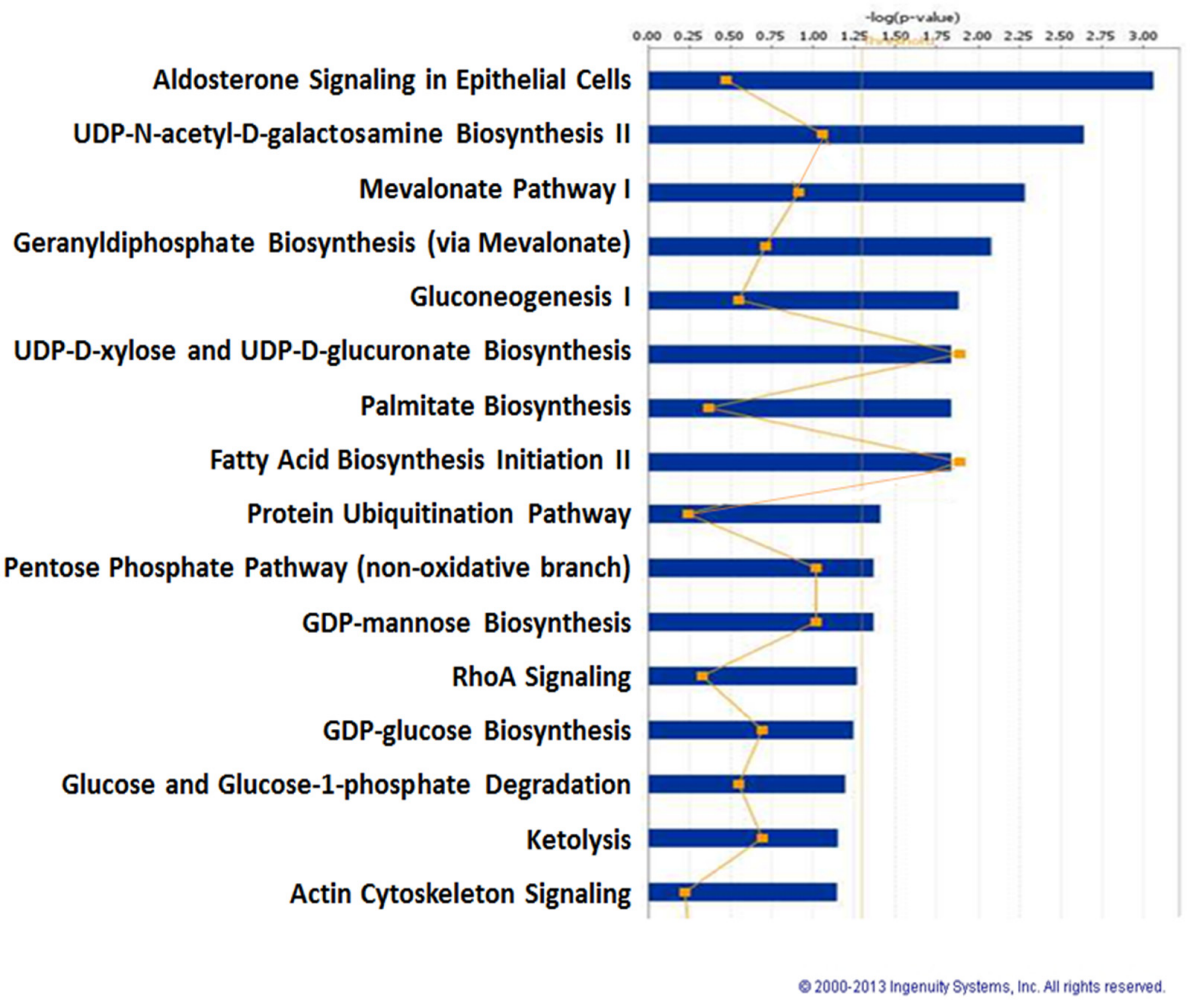
Top network functions generated using Ingenuity Pathway Analysis (IPA) for LNCaP prostate cancer cells treated with EBR. The graph represents host cell functions with the highest score (y-axis) based on the number of differentially regulated proteins. Reprinted from Ingenuity Pathways Analysis under a CC BY license, with permission from QIAGEN Silicon Valley, original copyright 2000–2013.

We next analyzed 160 differentially expressed proteins with respect to biological function, pathway and network using IPA software. As shown in Fig 1, according to the biological function analysis, EBR-altered proteins have functions in cellular growth and proliferation (16.6%), cell death and survival (14%), cellular assembly and organization (13.3%), cellular function and maintenance (13.3%), nucleic acid metabolism (5%), DNA replication (4%), protein synthesis and protein folding (3.7 and 1%, respectively) (Fig 1). The analysis of canonical pathways (p ≤ 0.05) identified aldosterone signaling in epithelial cells as well as cellular metabolic pathways (including UDP-N-acetyl-d-galactosamine biosynthesis, gluconeogenesis, fatty acid biosynthesis, protein ubiquitination pathway, cytoskeleton signaling and others) (Fig 2). CALR exhibited a significant alteration after 12 h EBR treatment with score 150, 11 matches, a heavy/light ratio of 0.4372 and 4 peptides (S1 Table). In addition according to the molecules associated with the SILAC analysis results CALR was downregulated by 2.287 compared to control samples (Table 1).

**Table 1:** Fold changes in the molecules associated with the SILAC analysis.

Symbol	Entrez Gene Name	Accession number GenPept/UniProt/Swiss-Prot	Fold Change
SCN11A	sodium channel, voltage-gated, type XI, alpha subunit	Q9UI33	7.059
PTDSS1	phosphatidylserine synthase 1	P48651	3.950
CACNA1S	calcium channel, voltage-dependent, L type, alpha 1S subunit	Q13698	3.395
HSPD1	heat shock 60 kDa protein 1 (chaperonin)	P10809	2.855
RNASEL	ribonuclease L (2',5'-oligoisoadenylate synthetase-dependent)	Q05823	2.329
HIST3H2BB	histone cluster 3, H2bb	Q8N257	-2.017
HSP90B1	heat shock protein 90kDa beta (Grp94), member 1	P14625	-2.113
EIF5B	eukaryotic translation initiation factor 5B	O60841	-2.235
**CALR**	**calreticulin Score: 150 Match: 11 # Peptides: 4**	**P27797**	**-2.287**
TUBB	tubulin, beta class I	P07437	-2.295
HSPA9	heat shock 70kDa protein 9 (mortalin)	P38646	-2.300
GANAB	glucosidase, alpha; neutral AB	Q14697	-2.316
AMPD3	adenosine monophosphate deaminase 3	Q01432	-2.350
KCNA5	potassium voltage-gated channel, shaker-related subfamily, member 5	P22460	-2.353
VDAC1	voltage-dependent anion channel 1	P21796	-2.478
ACADVL	acyl-CoA dehydrogenase, very long chain	P49748	-2.511
PSME1	proteasome (prosome, macropain) activator subunit 1 (PA28 alpha)	Q06323	-2.662
RASAL1	RAS protein activator like 1 (GAP1 like)	O95294	-2.766
PPARD	peroxisome proliferator-activated receptor delta	Q03181	-3.176
HSD17B2	hydroxysteroid (17-beta) dehydrogenase 2	P37059	-3.311

Altered members of the proteome were evaluated after EBR treatment.

Calreticulin was significantly decreased after 12 h EBR treatment according to the following data: calculated score: 150, matches: 11, heavy/light ratio: 0.4372 and number of peptides: 4.

### EBR-induced modulation of CALR expression

**Fig 3:**
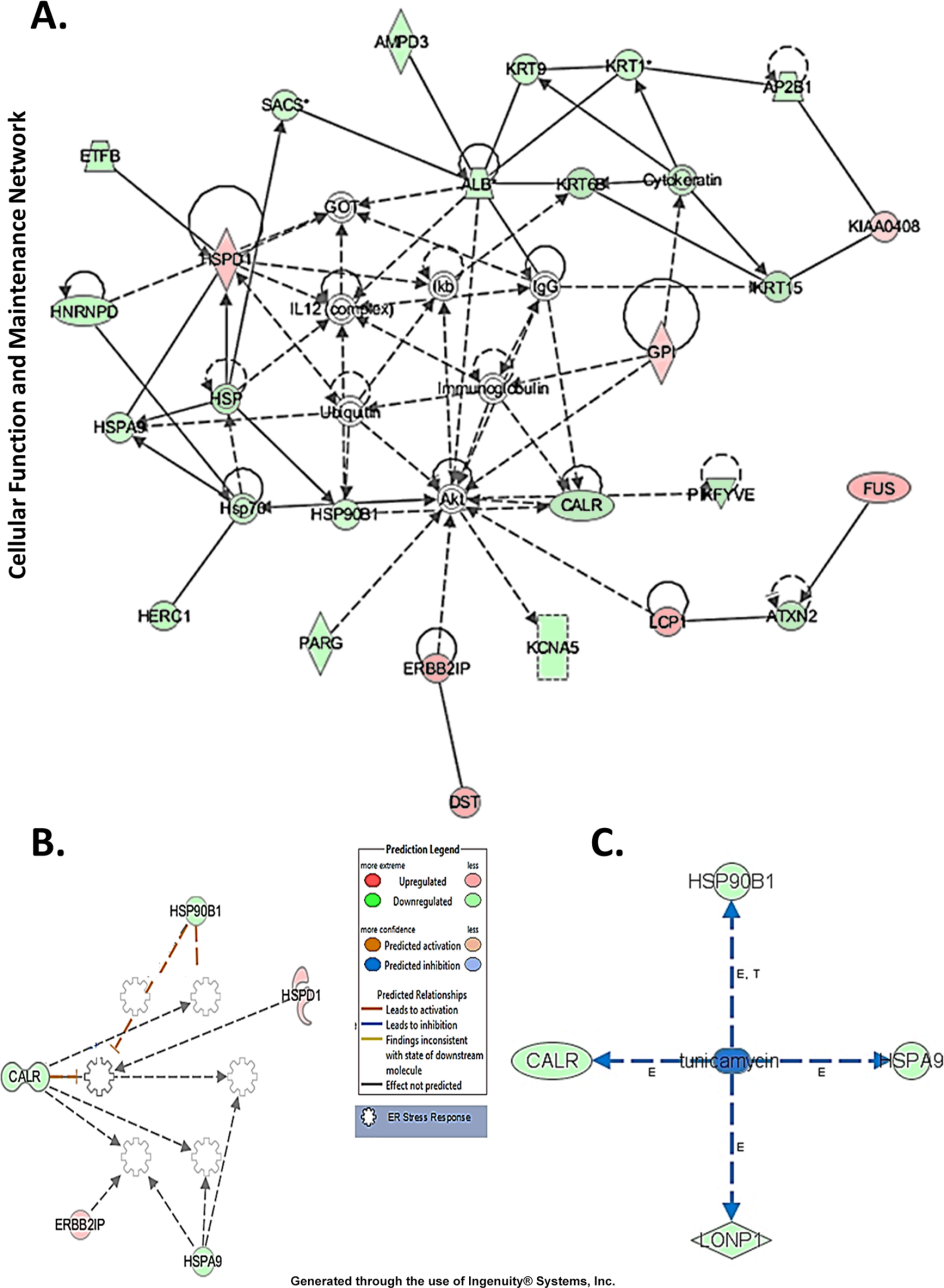
Ingenuity Pathway Analysis of proteins that were significantly altered after 12-h EBR treatment in LNCaP cells. A) Red, up-regulated proteins; green, significantly downregulated proteins; white, proteins known to be in the network but not identified in our study. The color depth indicates the magnitude of the change in protein expression levels. The shapes are indicative of the molecular class (i.e., protein family). B) IPA showing the pathways might involve in EBR-induced cellular alterations, **C.** tunicamycin, as an anticancer agent causing a similar effect. Lines connecting the molecules indicate molecular relationships. The arrow styles indicate specific molecular relationships and the directionality of the interaction. Reprinted from Ingenuity Pathways Analysis under a CC BY license, with permission from QIAGEN Silicon Valley, original copyright 2000–2013.

Differentially expressed proteins following EBR treatment were mapped to 7 specific functional networks with each network containing 11 or more “focus” members. The networks of interest corresponded to cell death and survival, cellular assembly and organization, cellular compromise, cellular function and maintenance, drug metabolism, lipid metabolism, cell morphology and cellular function. CALR was found a major protein in the cellular response, cellular assembly and organization networks (Fig 3A). Because CALR is a chaperone protein and because alterations in the CALR expression lead to the unfolded protein response and ER stress, we detected the interactions of CALR with other molecules to detect evidence of the UPR following EBR treatment in LNCaP prostate cancer cells. While we run IPA for pathway analysis after EBR treatment, the system predicted that heat shock chaperone protein family might also been involved in EBR-induced stress conditions as shown in Fig 3B. In addition, the IPA comparison of the EBR effect with other known anticancer agents causing similar effects indicated that EBR may act as an ER stress inducer like tunicamycin and is able to alter heat shock proteins and CALR (Fig 3C).

### Validation of protein identification and quantification

**Fig 4:**
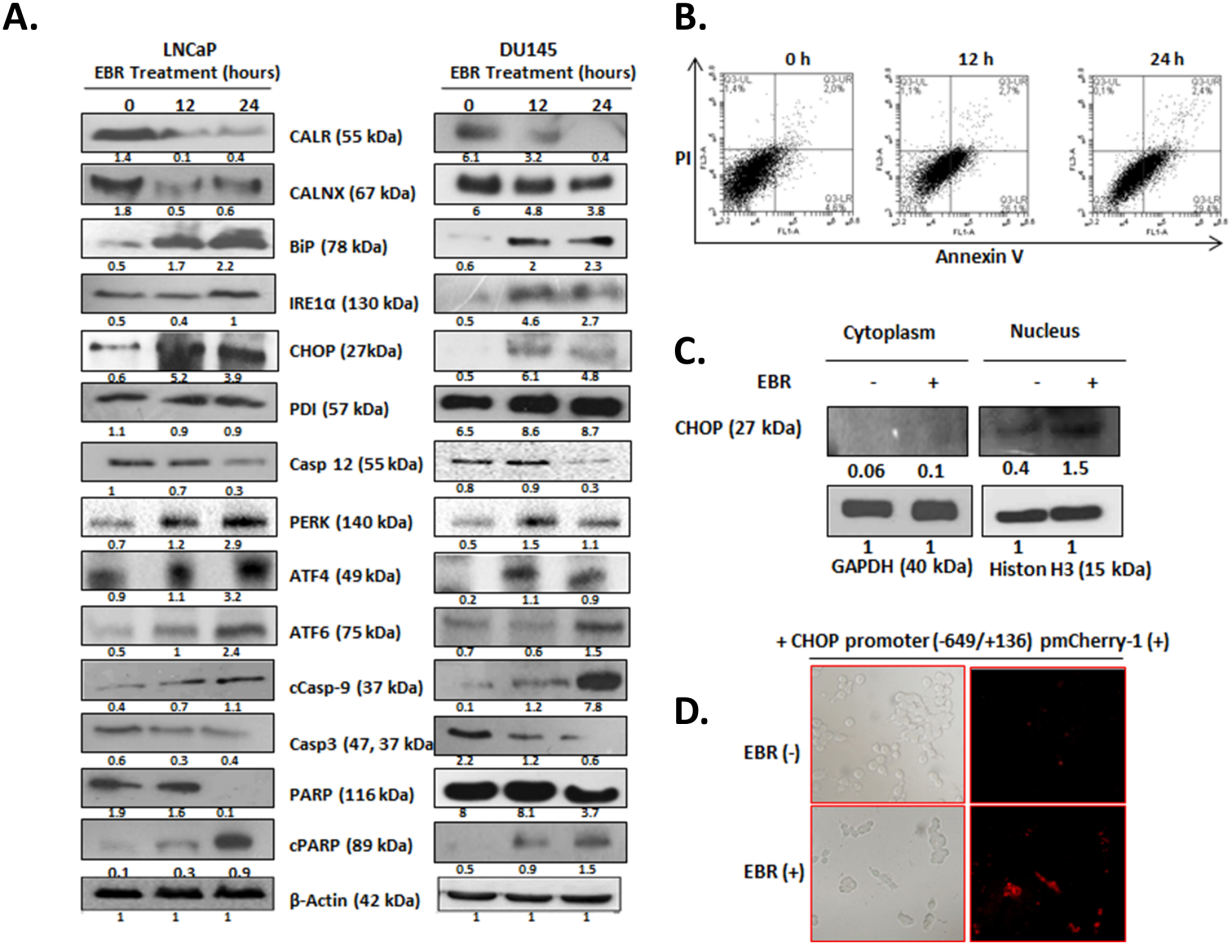
EBR induced ER stress and apoptosis. A) Total protein was isolated after EBR treatment, and the expression profiles of ER stress biomarkers, caspases and PARP cleavage were determined by immunoblotting using appropriate antibodies. β-actin was used as a loading control. B) Approximately 2 x 10^5^ cells were seeded into a six-well plate and treated with EBR for 12 and 24 h. Annexin V–PI staining was performed to determine apoptotic cell populations. Fluorescence signals from Annexin V–FITC and from PI are reported on the x-axis and y-axis, respectively. Numbers presented in the four quadrants represent the percentage of viable (lower left), necrotic (upper left), early apoptotic (lower right) and late apoptotic (upper right) cells. C) Following 12-h EBR treatment, cytosolic and nuclear proteins were isolated and separated in a 12% SDS gel, transferred onto a PVDF membrane and blotted with an anti-CHOP antibody. D) LNCaP cells were transfected with the reporter construct CHOP promoter (-649/+136) pmCherry-1. The CHOP activation was visualized with fluorescence microscopy. Excitation: 575 nm Emission: 601.

Because the protein interaction network and pathway analysis revealed the alteration of the UPR response in prostate cancer cells exposed to EBR, we next determined the expression levels of other UPR proteins by western blotting. We confirmed that the changes in the ratios of CALR in LNCaP cells were consistent with the ones derived from SILAC studies (Fig 4A, left panel). As shown in Fig 4, although EBR treatment downregulated the expression level of CALNX, the molecular chaperone BiP was upregulated. An excessive accumulation of misfolded proteins in ER triggers the dissociation of BiP from ER membrane-located receptors such as IRE1α, PERK and ATF6 as a significant ER stress response. In agreement with the altered BiP expression profile, IRE1α, PERK and ATF6 were upregulated following EBR treatment in LNCaP cells (Fig 4A, left panel). We also determined the expression levels of other ER stress-related proteins such as CHOP, ATF4 and PDI in the total protein lysates. We observed that the exposure of LNCaP cells to EBR increased the expression of CHOP in both cytoplasmic and nuclear fractions (Fig 4C). Interestingly, PDI, a stress protein abundant in ER, did not elevated in response to EBR treatment. Excessive and prolonged ER stress triggers apoptosis. Consistent with this finding, EBR time-dependently activated caspase-12, caspase-9 and caspase-3 and induced the cleavage of PARP (Fig 4A, left panel). Similar results were observed in DU145 prostate cancer cells exposed to EBR (Fig 4A, right panel). Annexin-V/PI staining results confirmed that EBR induced apoptosis in LNCaP cells (Fig 4B). These results indicate that EBR induced apoptosis via the UPR axis in both prostate cancer cells regardless of the functional AR expression.

**Fig 5:**
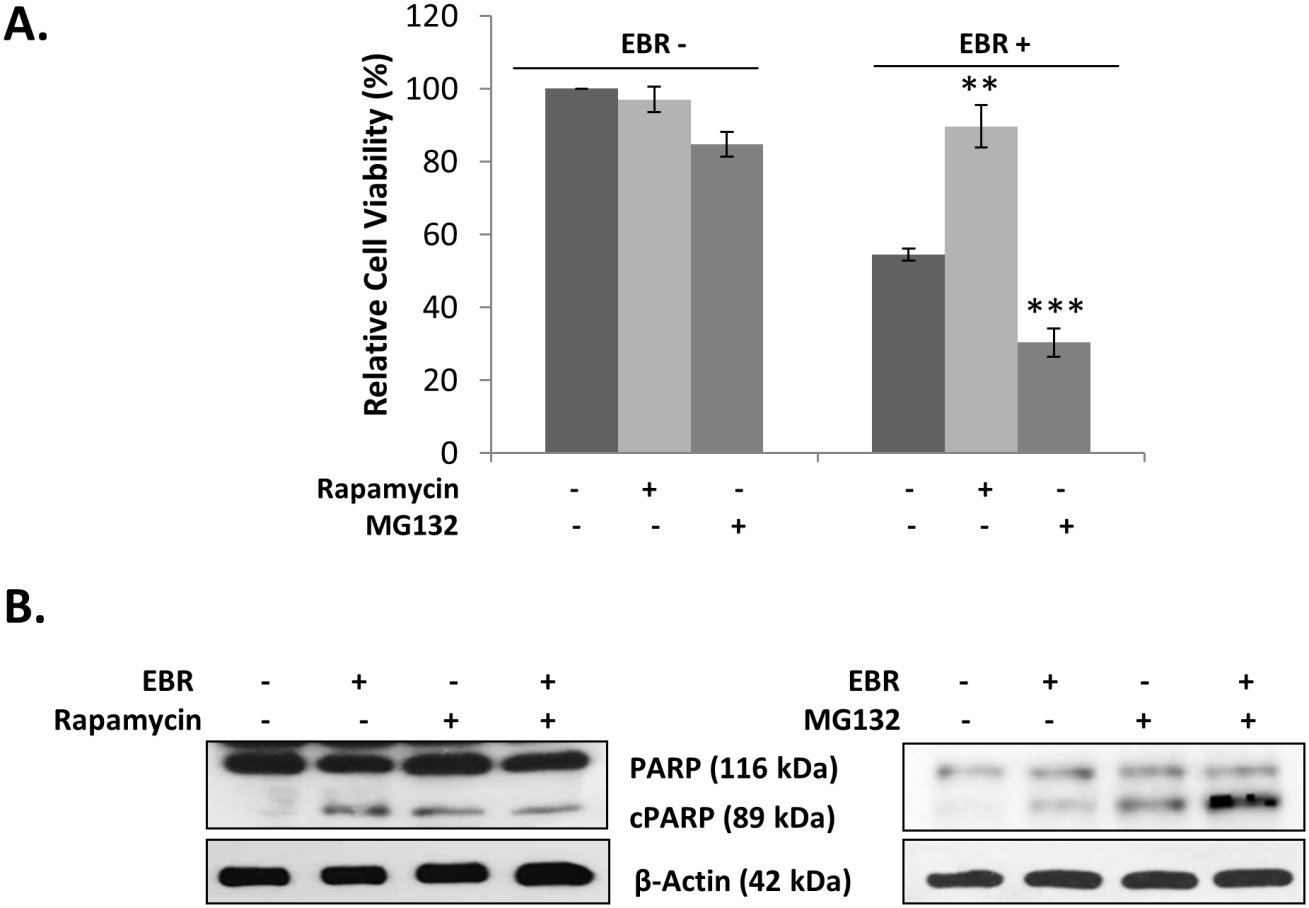
EBR-induced apoptosis was ER stress-dependent. A) Cell viability loss following co-treatment with rapamycin (10 nM) or pre-treatment with MG132 (10 μM) in the presence of EBR (25 μM) for 12 h was measured by MTT assay. B) The effect of the rapamycin or MG132 +/- EBR was determined by PARP immunoblotting. β-actin was used as a loading control.

To verify that the result of EBR-induced apoptosis was related to the UPR, LNCaP cells were transfected with the reporter construct of CHOP promoter (-649/+136) pmCherry-1 plasmid. As shown in Fig 4D, EBR clearly induced CHOP activity and the resulting puncta pattern in LNCaP cells. To further validate the effect of EBR on ER stress-related apoptosis, we inhibited mTOR by rapamycin treatment to block *de novo* mRNA and protein synthesis. Inhibition of mRNA synthesis leads to the accumulation of unfolded/misfolded proteins in the ER and therefore can potentially alleviate ER stress-induced cell death [12]. We observed that co-treatment with rapamycin significantly prevented EBR-induced cell viability loss (Fig 5A) and apoptosis (Fig 5B, left panel) in LNCaP cells. In contrary, 26S proteasome inhibitor MG132 co-treatment further increased cell viability loss (Fig 5A) and apoptosis (Fig 5B, right panel). According to the obtained data, we suggest that the presence of MG132 prevented the EBR-induced degradation of misfolded proteins and exacerbated the apoptotic response in LNCaP prostate cancer cells. Collectively, these results support the hypothesis that the EBR-induced cell death mechanism is mediated by the UPR in prostate cancer cells.

### CALR is an important target of EBR

**Fig 6:**
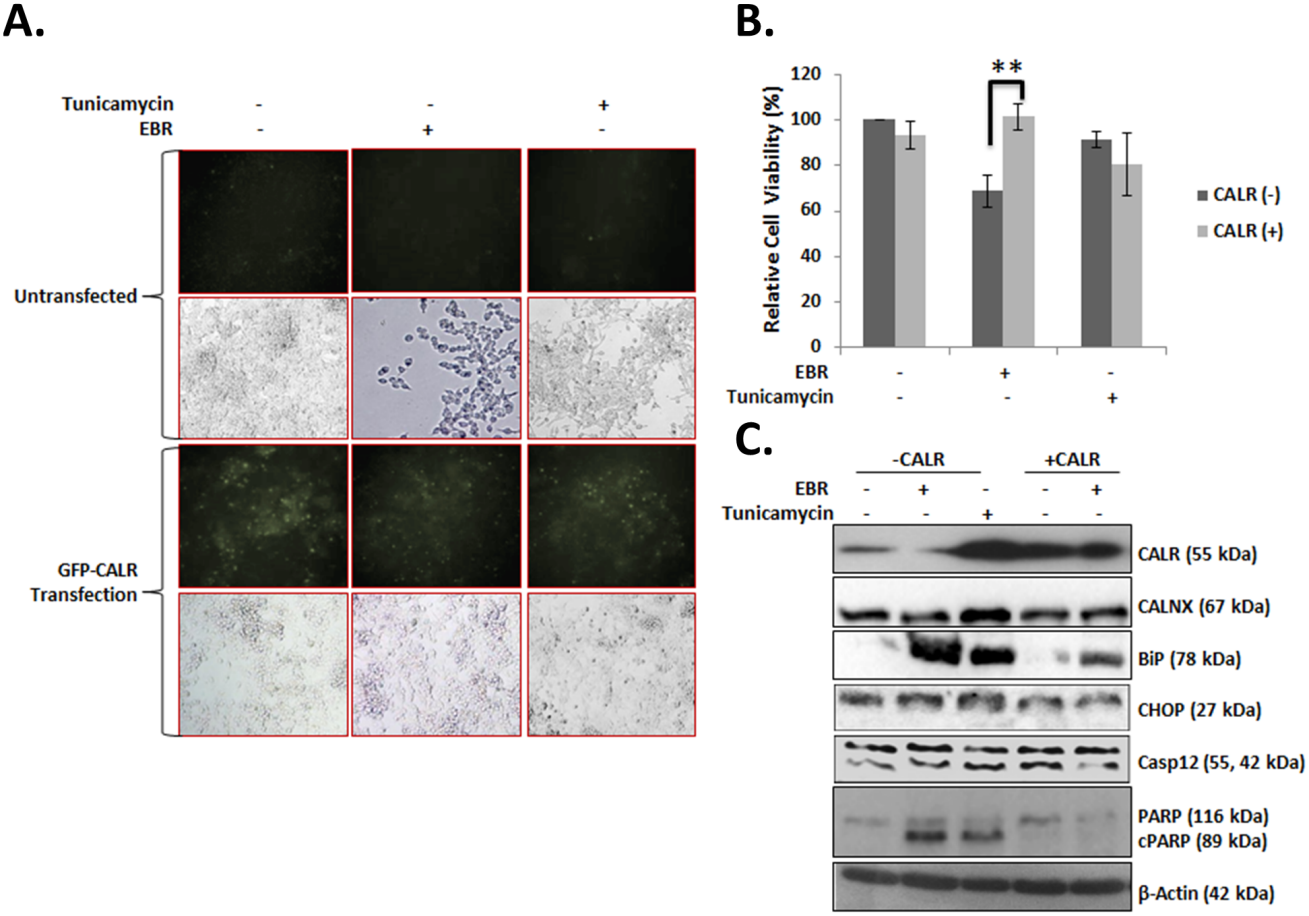
CALR was an important target of EBR treatment. A) The effect of 12-h EBR treatment after pEGFP-CALR plasmid transfection was visualized via GFP dots in LNCaP cells. Tunicamycin treatment was performed as a positive control. B) The effect of EBR in pEGFP-CALR plasmid-transfected LNCaP cells following 12-h EBR treatment was determined by MTT assay. C) The effect of EBR on ER stress and apoptosis biomarkers in CALR-transfected LNCaP cells was determined by immunoblotting. Tunicamycin-treated cell lysates were used as a positive control; β-actin was used as a loading control.

To begin to understand the molecular mechanism behind EBR-induced UPR, we transiently transfected LNCaP cells with a GFP-tagged pCMV-CALR plasmid (Fig 6A). Concomitantly we utilized tunicamycin as a positive control to activate ER stress in AR-sensitive LNCaP cells. Although CALR overexpression prevented the EBR-dependent loss of cell viability, it did not exert same effect following tunicamycin treatment (Fig 6B). Although tunicamycin activated the ER stress pathway by upregulating the expression of CALR, CALNX, BiP and CHOP, EBR treatment only induced the upregulation of CHOP but downregulated CALR and CALNX. CALR overexpression diminished the potential effect of EBR on ER signaling players, which indicated that CALR is a critical target of EBR in LNCaP cells. In addition, we suggest that EBR differs from tunicamycin through activating different targets of the ER stress machinery (Fig 6C).

**Fig 7:**
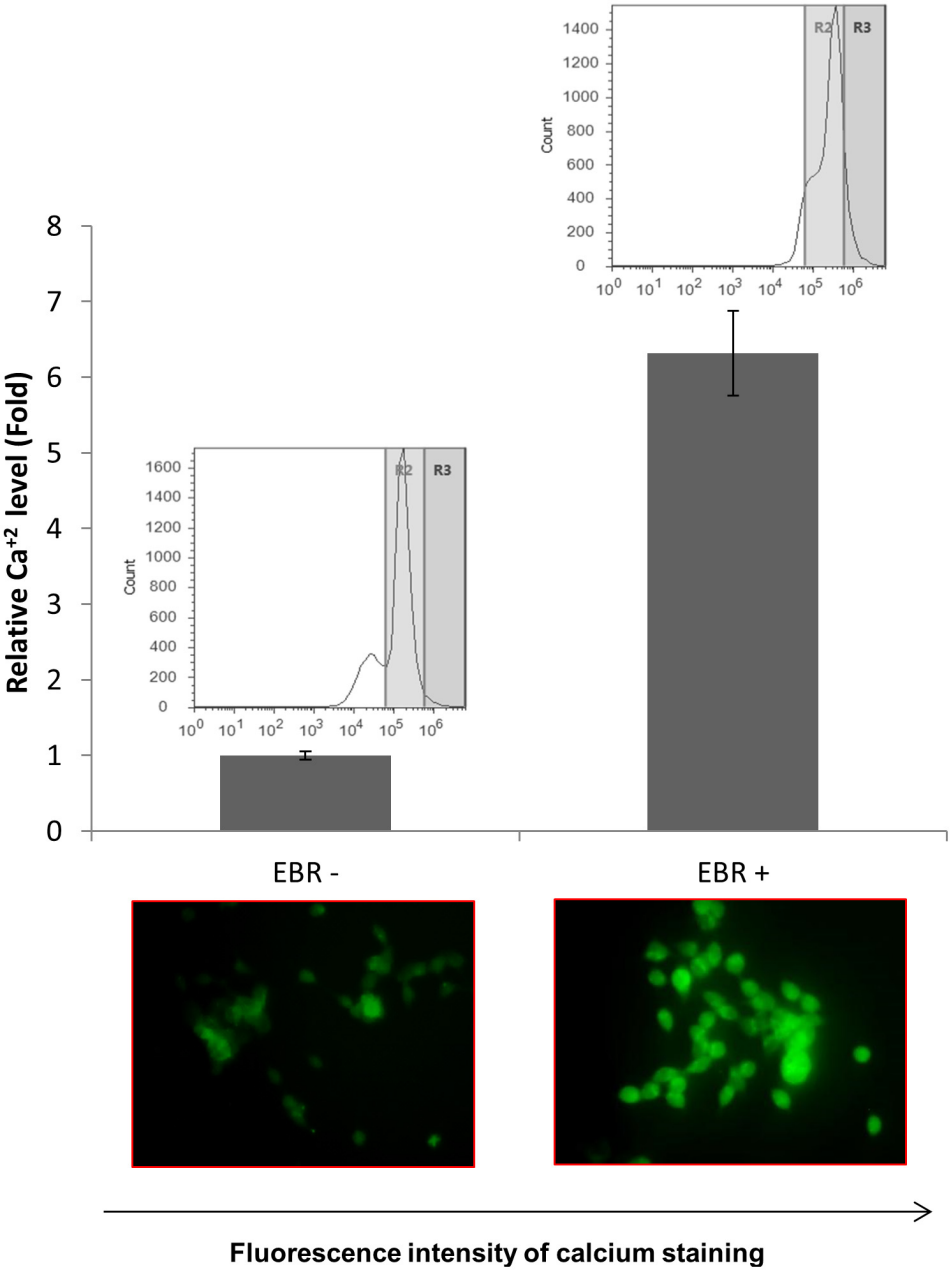
Ca^2+^ levels were altered following EBR treatment. Cells were treated with EBR for 12 h and stained with calcium green. The fluorescence intensity was determined with FACS flow and fluorescence microscopy (excitation 506 nm, emission: 531 nm).

Next, we determined the apoptotic efficiency of EBR in both wt and CALR+ LNCaP cells. As shown in Fig 6C, EBR activated caspase-12, which culminated in PARP cleavage in LNCaP wt cells but not in CALR+ LNCaP cells (Fig 6C). As CALR is an important regulator of intracellular Ca^2+^ buffering capacity, which also triggers apoptosis, we examined Ca^2+^ levels following EBR treatment (Fig 7). We observed that EBR treatment increase the release of Ca^2+^ by 6-fold in LNCaP cells.

**Fig 8:**
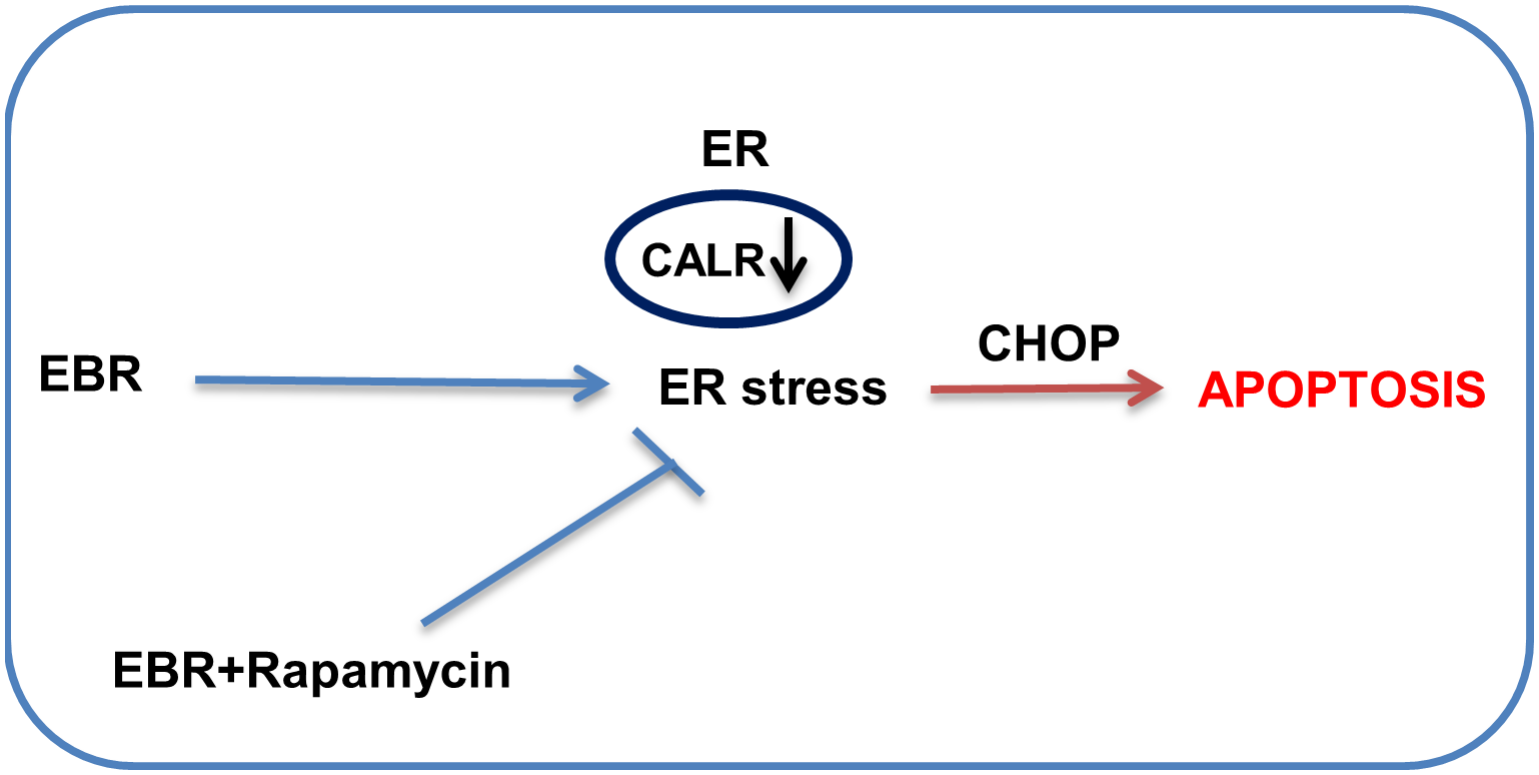
ER stress is involved in EBR-induced apoptosis in prostate cancer cells.

Finally, we proposed that EBR induced apoptosis in prostate cancer cells by causing ER stress related to CALR downregulation and Ca^2+^ release into the cytosol (Fig 8).

## Discussion

EBR, a plant growth regulator, has been recently suggested as a candidate chemotherapeutic drug because of its ability to induce cell cycle arrest and apoptosis in different cancer cell lines without affecting normal epithelial cells [4]. Given that EBR-induced apoptosis is more effective in NHR-expressing cells than in non-NHR-expressing cells, it has been suggested that its steroid-like structure acts on NHRs and triggers apoptosis [3]. However, the clear apoptotic effect on prostate or breast cancer cell lines with non-functional NHR, such as DU 145 or MDA-MB-231 cells [4, 5], indicated an unknown common target for apoptosis. Although there are reports suggesting that different cellular mechanisms are involved in EBR-induced apoptosis, little is known about its mechanistic action. Therefore, in our study, we were focused on the idea of proposing a mutual target using a global approach.

Proteomic studies are powerful approaches to examine the molecular mechanisms of chemotherapeutic drugs and their interacting signaling mechanisms. Therefore, to clarify the apoptotic effect of EBR through evaluating changes in the proteome, we performed SILAC-based mass spectrometry analysis in LNCaP cells. Our study indicated that 160 different proteins exhibited significant alterations after 12 h EBR treatment (S1 Table). According to our biological function data, those proteins played roles in cellular growth and proliferation (16.6%), cell death and survival (14%), cellular assembly and organization (13.3%), cellular function and maintenance (13.3%), nucleic acid metabolism (5%), DNA replication (4%), protein synthesis (3.7%) and protein folding (1%) (Fig 1). In addition, the pathway analysis revealed that aldosterone signaling, gluconeogenesis, fatty acid biosynthesis, the protein ubiquitination pathway and cytoskeleton signaling were affected (Fig 2). The proteins in the cell death and survival, cellular function and maintenance pathways were also mapped (Fig 3A and 3B). Among the significantly altered proteins following EBR treatment, CALR was noteworthy based on the following parameters: score: 150, matches: 11, heavy/light ratio: 0.4372 and number of peptides: 4 according to the MDM analysis (Fig 4A). The significant downregulation of CALR prompted us to investigate the potential role of EBR-induced ER stress. CALR, as an ER chaperone, plays role in the folding process of newly synthesized proteins as well as in the decoding of both normal and pathological Ca^2+^ signals due to its buffering capacity [13]. Downregulation of CALR has been shown to lead to rapid and severe alterations in ER Ca^2+^ homeostasis [14]. CALR, as an androgen-responsive gene, has been shown to be downregulated in castrated rat prostate or *in vitro* models [15]. CALR binding to misfolded proteins prevents their export from the ER lumen to the cytosol. In addition, CALR antisense oligonucleotides significantly increase the sensitivity of LNCaP cells to Ca^2+^ ionophore A23187-induced cell death [16]. Together, these findings validated the approach from SILAC analysis that EBR might be effective in triggering ER stress to induce apoptosis. In addition, when we investigated the CALR-related pathways using IPA, we detected that the downregulation of CALR could be related to ER stress. Therefore, we confirmed our results by western blotting. As shown in Fig 4A, EBR decreased the CALR and CALNX expression profiles time-dependently by causing caspase-dependent apoptotic cell death in both AR-expressing and AR-non-expressing prostate cancer cells (Fig 4A and 4B). In addition, the levels of BiP and CHOP in total lysates and CHOP translocation to the nucleus were upregulated (Fig 4C), suggesting that EBR is a candidate to induce ER stress. The effect of EBR was also confirmed by the transfection of the CHOP promoter (-649/+136) tagged with pmCherry-1, suggesting that EBR is a candidate to induce ER stress (Fig 4D). The upregulation in BiP is crucial to initiate the ER stress response via ER membrane-located proteins such as IRE1α, PERK and ATF6 which in turn activates XBP1, ATF4 and ATF6 itself as transcription factors to induce ER stress. CHOP expression [17]. Particulary, CHOP expression is induced in response to XBP1 transactivation. Consistent with this fact, EBR treatment was also found to upregulate IRE1α and CHOP expression in LNCaP prostate cancer cells. Once CHOP is expressed, it can trigger the expression of pro-apoptotic proteins, acts on the mitochondrial membrane to release cytochrome c, and triggers the caspase cascade *via* caspase-9 and caspase-3 [18, 19]. Many agents inducing apoptosis via ER-stress have been shown to upregulate IRE1 family members and the downstream targets XBP1 and CHOP [20]. Caspase-12, caspase-9, caspase-3 and PARP cleavage profiles supported our hypothesis (Fig 4A).

To clarify the mechanism of EBR-activated ER stress, we suppressed *de novo* protein synthesis through mTOR complex inhibition by rapamycin [21]. Rapamycin is a well-known translational inhibitor that has been shown to prevent tunicamycin- and bortezomib-induced ER-stress in MEF and Elt3 cells, respectively [22, 23]. Similar to this finding, we found that rapamycin co-treatment prevented EBR-induced apoptotic cell death (Fig 5A and 5B). In contrary, inhibition of proteasomal degradation by MG132 further induced EBR-induced apoptosis. Supporting this finding, recent data suggested that inhibition of proteasomal degradation resulted in a quick apoptotic induction response in proliferating cells [24].

Recently, a number of reports have suggested that CALR is a fine-tuning target to modulate cell survival and death-related signaling pathways. According to the expression level of CALR, protein tyrosine kinases or phosphatases might be altered to decide cell fate under apoptotic stimuli. CALR overexpression was suggested as a promoting factor in differentiation-induced apoptosis in H9c2 embryonic rat heart cells under trans-retinoic acid stimuli via modulating the Akt signaling cascade [25]. However, the overexpression of CALR might prevent drug-induced apoptosis via the enhanced buffering potential of Ca^2+^ [26]. Increased cellular Ca^2+^ influx is a mediator of apoptosis and should be sensed and corrected by CALR. In this study, CALR overexpression abolished the cytotoxic effect of EBR by preventing EBR-induced BiP and CHOP upregulation, leading to the ER-dependent apoptosis cascade. This result was also confirmed by caspase-12 and PARP cleavage profiles. Therefore, we suggest that EBR might act on a Ca^2+^ buffering mechanism via altering CALR expression regardless of NHR status in prostate cancer cells. Increased Ca^2+^ levels, which was shown by calcium green staining, was observed following EBR treatment.

In conclusion, all data in this study suggest that EBR is an effective apoptotic agent through modulating the CALR expression profile and thus causing deficient Ca^2+^ buffering potential regardless of NHR expression in prostate cancer cells (Fig 8). In addition, this study is the first to present the proteomic alterations due to EBR treatment investigated by SILAC assay, which also presents other novel targets for EBR.

## Supporting Information

S1 Table One hundred sixty significantly altered proteins after 12 h EBR treatment identified with SILAC LC-MS/MS. (DOCX)
