# Supplementary material for: SILAC-Based Mass Spectrometry Analysis Reveals That Epibrassinolide Induces Apoptosis via Activating Endoplasmic Reticulum Stress in Prostate Cancer Cells
Source: PLoS One. 2015 Sep 9;10(9):e0135788. doi: 10.1371/journal.pone.0135788 (PMC4564160; doi:10.1371/journal.pone.0135788)
Supplement: S1 Table — (DOCX) [file pone.0135788.s001.docx]

**S1 Table. One hundred sixty significantly altered proteins after 12 h EBR treatment identified with SILAC LC-MS/MS.**

| **Accession Nr** | **Accession** | **Name** |  | | |  |
| --- | --- | --- | --- | --- | --- | --- |
|  |  |  | **Score** | **Matches** | **Heavy /**  **Light**  **Ratio** | **# Peptides** |
| [P42356](http://www.uniprot.org/uniprot/P42356) | PI4KA_HUMAN | Phosphatidylinositol 4-kinase alpha | 20 | 23 | 14,6089 | 2 |
| [P55795](http://www.uniprot.org/uniprot/P55795) | HNRH2_HUMAN | Heterogeneous nuclear ribonucleoprotein H2 | 34 | 6 | 10,5784 | 2 |
| [A6NHN0](http://www.uniprot.org/uniprot/A6NHN0) | OTOL1_HUMAN | Otolin-1 | 15 | 10 | 6,41011 | 2 |
| [Q6RI45](http://www.uniprot.org/uniprot/Q6RI45) | BRWD3_HUMAN | Bromodomain and WD repeat-containing protein 3 | 23 | 8 | 3,73151 | 2 |
| [O14879](http://www.uniprot.org/uniprot/O14879) | IFIT3_HUMAN | Interferon-induced protein with tetratricopeptide repeats 3 | 22 | 5 | 3,5834 | 2 |
| [Q5VU92](http://www.uniprot.org/uniprot/Q5VU92) | DC121_HUMAN | DDB1- and CUL4-associated factor 12-like protein 1 | 21 | 8 | 3,47064 | 1 |
| [Q13698](http://www.uniprot.org/uniprot/Q13698) | CAC1S_HUMAN | Voltage-dependent L-type calcium channel subunit alpha-1S | 17 | 10 | 3,39515 | 1 |
| [Q03001](http://www.uniprot.org/uniprot/Q03001) | DYST_HUMAN | Dystonin | 13 | 80 | 3,33049 | 2 |
| [Q8WUQ7](http://www.uniprot.org/uniprot/Q8WUQ7) | CATIN_HUMAN | Cactin | 26 | 9 | 3,05615 | 1 |
| [P06744](http://www.uniprot.org/uniprot/P06744) | G6PI_HUMAN | Glucose-6-phosphate isomerase | 151 | 11 | 3,03238 | 3 |
| [Q96RT1](http://www.uniprot.org/uniprot/Q96RT1) | LAP2_HUMAN | Protein LAP2 | 23 | 7 | 2,94218 | 1 |
| [Q96Q05](http://www.uniprot.org/uniprot/Q96Q05) | TPPC9_HUMAN | Trafficking protein particle complex subunit 9 | 18 | 9 | 2,86758 | 1 |
| [P10809](http://www.uniprot.org/uniprot/P10809) | CH60_HUMAN | 60 kDa heat shock protein, mitochondrial | 63 | 19 | 2,85485 | 1 |
| [Q8TE60](http://www.uniprot.org/uniprot/Q8TE60) | ATS18_HUMAN | A disintegrin and metalloproteinase with thrombospondin motifs 18 | 21 | 8 | 2,82165 | 1 |
| [Q9H267](http://www.uniprot.org/uniprot/Q9H267) | VP33B_HUMAN | Vacuolar protein sorting-associated protein 33B | 26 | 10 | 2,80976 | 1 |
| [Q9BQS8](http://www.uniprot.org/uniprot/Q9BQS8) | FYCO1_HUMAN | FYVE and coiled-coil domain-containing protein 1 | 14 | 16 | 2,72347 | 1 |
| [P13473](http://www.uniprot.org/uniprot/P13473) | LAMP2_HUMAN | Lysosome-associated membrane glycoprotein 2 | 60 | 2 | 2,62403 | 2 |
| [Q14974](http://www.uniprot.org/uniprot/Q14974) | IMB1_HUMAN | Importin subunit beta-1 | 28 | 8 | 2,5556 | 1 |
| [Q6PI48](http://www.uniprot.org/uniprot/Q6PI48) | SYDM_HUMAN | Aspartate--tRNA ligase, mitochondrial | 16 | 10 | 2,35927 | 1 |
| [Q05823](http://www.uniprot.org/uniprot/Q05823) | RN5A_HUMAN | 2-5A-dependent ribonuclease | 29 | 12 | 2,32912 | 1 |
| [O95613](http://www.uniprot.org/uniprot/O95613) | PCNT_HUMAN | Pericentrin | 15 | 37 | 2,22179 | 1 |
| [P33176](http://www.uniprot.org/uniprot/P33176) | KINH_HUMAN | Kinesin-1 heavy chain | 66 | 16 | 2,19983 | 2 |
| [O15014](http://www.uniprot.org/uniprot/O15014) | ZN609_HUMAN | Zinc finger protein 609 | 21 | 13 | 2,18461 | 2 |
| [P29401](http://www.uniprot.org/uniprot/P29401) | TKT_HUMAN | Transketolase | 38 | 6 | 2,17058 | 2 |
| [Q562R1](http://www.uniprot.org/uniprot/Q562R1) | ACTBL_HUMAN | Beta-actin-like protein 2 | 199 | 15 | 2,16902 | 5 |
| [Q96DF8](http://www.uniprot.org/uniprot/Q96DF8) | DGC14_HUMAN | Protein DGCR14 | 22 | 9 | 2,16595 | 2 |
| [Q12766](http://www.uniprot.org/uniprot/Q12766) | HMGX3_HUMAN | HMG domain-containing protein 3 | 27 | 6 | 2,16317 | 1 |
| [P13796](http://www.uniprot.org/uniprot/P13796) | PLSL_HUMAN | Plastin-2 | 48 | 9 | 2,15539 | 2 |
| [Q8WUJ3](http://www.uniprot.org/uniprot/Q8WUJ3) | K1199_HUMAN | Protein KIAA1199 | 21 | 9 | 2,12168 | 2 |
| [Q9UQ35](http://www.uniprot.org/uniprot/Q9UQ35) | SRRM2_HUMAN | Serine/arginine repetitive matrix protein 2 | 20 | 27 | 2,10375 | 2 |
| [Q04609](http://www.uniprot.org/uniprot/Q04609) | FOLH1_HUMAN | Glutamate carboxypeptidase 2 | 100 | 20 | 2,04907 | 5 |
| [P55072](http://www.uniprot.org/uniprot/P55072) | TERA_HUMAN | Transitional endoplasmic reticulum ATPase | 200 | 15 | 2,04561 | 3 |
| [Q96QT4](http://www.uniprot.org/uniprot/Q96QT4) | TRPM7_HUMAN | Transient receptor potential cation channel subfamily M member 7 | 17 | 17 | 2,03277 | 1 |
| [P51149](http://www.uniprot.org/uniprot/P51149) | RAB7A_HUMAN | Ras-related protein Rab-7a | 67 | 6 | 0,49783 | 1 |
| [Q8N257](http://www.uniprot.org/uniprot/Q8N257) | H2B3B_HUMAN | Histone H2B type 3-B | 133 | 8 | 0,49571 | 1 |
| [O00299](http://www.uniprot.org/uniprot/O00299) | CLIC1_HUMAN | Chloride intracellular channel protein 1 | 73 | 5 | 0,49458 | 2 |
| [P35237](http://www.uniprot.org/uniprot/P35237) | SPB6_HUMAN | Serpin B6 | 66 | 5 | 0,49303 | 4 |
| [P55084](http://www.uniprot.org/uniprot/P55084) | ECHB_HUMAN | Trifunctional enzyme subunit beta, mitochondrial | 30 | 12 | 0,49035 | 3 |
| [P14625](http://www.uniprot.org/uniprot/P14625) | ENPL_HUMAN | Endoplasmin | 104 | 13 | 0,47319 | 3 |
| [P22392](http://www.uniprot.org/uniprot/P22392) | NDKB_HUMAN | Nucleoside diphosphate kinase B | 62 | 5 | 0,47312 | 2 |
| [P55084](http://www.uniprot.org/uniprot/P55084) | ECHB_HUMAN | Trifunctional enzyme subunit beta, mitochondrial | 62 | 15 | 0,4694 | 2 |
| [P11047](http://www.uniprot.org/uniprot/P11047) | LAMC1_HUMAN | Laminin subunit gamma-1 | 127 | 15 | 0,46755 | 4 |
| [P49327](http://www.uniprot.org/uniprot/P49327) | FAS_HUMAN | Fatty acid synthase | 79 | 15 | 0,46492 | 1 |
| [O60701](http://www.uniprot.org/uniprot/O60701) | UGDH_HUMAN | UDP-glucose 6-dehydrogenase | 23 | 7 | 0,45813 | 1 |
| [Q9P243](http://www.uniprot.org/uniprot/Q9P243) | ZFAT_HUMAN | Zinc finger protein ZFAT | 13 | 16 | 0,45114 | 1 |
| [O60841](http://www.uniprot.org/uniprot/O60841) | IF2P_HUMAN | Eukaryotic translation initiation factor 5B | 26 | 16 | 0,4475 | 1 |
| [P19367](http://www.uniprot.org/uniprot/P19367) | HXK1_HUMAN | Hexokinase-1 | 19 | 23 | 0,44729 | 2 |
| [P48643](http://www.uniprot.org/uniprot/P48643) | TCPE_HUMAN | T-complex protein 1 subunit epsilon | 47 | 21 | 0,44294 | 1 |
| [P30048](http://www.uniprot.org/uniprot/P30048) | PRDX3_HUMAN | Thioredoxin-dependent peroxide reductase, mitochondrial | 82 | 3 | 0,43812 | 1 |
| [P27797](http://www.uniprot.org/uniprot/P27797) | CALR_HUMAN | Calreticulin | 150 | 11 | 0,4372 | 4 |
| [P07437](http://www.uniprot.org/uniprot/P07437) | TBB5_HUMAN | Tubulin beta chain | 47 | 11 | 0,4358 | 3 |
| [P38646](http://www.uniprot.org/uniprot/P38646) | GRP75_HUMAN | Stress-70 protein, mitochondrial | 63 | 11 | 0,43475 | 3 |
| [Q14697](http://www.uniprot.org/uniprot/Q14697) | GANAB_HUMAN | Neutral alpha-glucosidase AB | 23 | 6 | 0,43169 | 1 |
| [Q9Y266](http://www.uniprot.org/uniprot/Q9Y266) | NUDC_HUMAN | Nuclear migration protein nudC | 36 | 6 | 0,43041 | 2 |
| [P63010](http://www.uniprot.org/uniprot/P63010) | AP2B1_HUMAN | AP-2 complex subunit beta | 29 | 7 | 0,42738 | 1 |
| [Q96QD9](http://www.uniprot.org/uniprot/Q96QD9) | UIF_HUMAN | UAP56-interacting factor | 17 | 10 | 0,42585 | 2 |
| [Q01432](http://www.uniprot.org/uniprot/Q01432) | AMPD3_HUMAN | AMP deaminase 3 | 16 | 9 | 0,42546 | 1 |
| [Q14103](http://www.uniprot.org/uniprot/Q14103) | HNRPD_HUMAN | Heterogeneous nuclear ribonucleoprotein D0 | 48 | 5 | 0,42539 | 1 |
| [Q01484](http://www.uniprot.org/uniprot/Q01484) | ANK2_HUMAN | Ankyrin-2 | 29 | 30 | 0,42513 | 2 |
| [P22460](http://www.uniprot.org/uniprot/P22460) | KCNA5_HUMAN | Potassium voltage-gated channel subfamily A member 5 | 19 | 8 | 0,42498 | 1 |
| [Q8WZ42](http://www.uniprot.org/uniprot/Q8WZ42) | TITIN_HUMAN | Titin | 18 | 336 | 0,42498 | 1 |
| [P54868](http://www.uniprot.org/uniprot/P54868) | HMCS2_HUMAN | Hydroxymethylglutaryl-CoA synthase, mitochondrial | 72 | 3 | 0,42058 | 2 |
| [P38117](http://www.uniprot.org/uniprot/P38117) | ETFB_HUMAN | Electron transfer flavoprotein subunit beta | 91 | 9 | 0,41141 | 3 |
| [P49327](http://www.uniprot.org/uniprot/P49327) | FAS_HUMAN | Fatty acid synthase | 56 | 22 | 0,40963 | 1 |
| [Q96IX5](http://www.uniprot.org/uniprot/Q96IX5) | USMG5_HUMAN | Up-regulated during skeletal muscle growth protein 5 | 93 | 4 | 0,4059 | 2 |
| [Q8IZT6](http://www.uniprot.org/uniprot/Q8IZT6) | ASPM_HUMAN | Abnormal spindle-like microcephaly-associated protein | 16 | 47 | 0,40439 | 1 |
| [P21796](http://www.uniprot.org/uniprot/P21796) | VDAC1_HUMAN | Voltage-dependent anion-selective channel protein 1 | 60 | 5 | 0,40348 | 1 |
| [P49748](http://www.uniprot.org/uniprot/P49748) | ACADV_HUMAN | Very long-chain specific acyl-CoA dehydrogenase, mitochondrial | 38 | 11 | 0,3983 | 1 |
| [P48163](http://www.uniprot.org/uniprot/P48163) | MAOX_HUMAN | NADP-dependent malic enzyme | 92 | 10 | 0,39028 | 2 |
| [Q04609](http://www.uniprot.org/uniprot/Q04609) | FOLH1_HUMAN | Glutamate carboxypeptidase 2 | 151 | 20 | 0,38936 | 8 |
| [Q9NZJ4](http://www.uniprot.org/uniprot/Q9NZJ4) | SACS_HUMAN | Sacsin | 23 | 31 | 0,38448 | 1 |
| [Q06323](http://www.uniprot.org/uniprot/Q06323) | PSME1_HUMAN | Proteasome activator complex subunit 1 | 58 | 5 | 0,37569 | 2 |
| [Q6PID8](http://www.uniprot.org/uniprot/Q6PID8) | KLD10_HUMAN | Kelch domain-containing protein 10 | 17 | 7 | 0,36947 | 1 |
| [P13473](http://www.uniprot.org/uniprot/P13473) | LAMP2_HUMAN | Lysosome-associated membrane glycoprotein 2 | 49 | 2 | 0,36739 | 2 |
| [Q969H8](http://www.uniprot.org/uniprot/Q969H8) | CS010_HUMAN | UPF0556 protein C19orf10 | 56 | 1 | 0,36374 | 1 |
| [O95294](http://www.uniprot.org/uniprot/O95294) | RASL1_HUMAN | RasGAP-activating-like protein 1 | 40 | 11 | 0,36156 | 2 |
| [O00754](http://www.uniprot.org/uniprot/O00754) | MA2B1_HUMAN | Lysosomal alpha-mannosidase | 48 | 7 | 0,34792 | 1 |
| [P21333](http://www.uniprot.org/uniprot/P21333) | FLNA_HUMAN | Filamin-A | 20 | 31 | 0,34078 | 1 |
| [Q9ULL4](http://www.uniprot.org/uniprot/Q9ULL4) | PLXB3_HUMAN | Plexin-B3 | 25 | 11 | 0,33878 | 1 |
| [Q15751](http://www.uniprot.org/uniprot/Q15751) | HERC1_HUMAN | Probable E3 ubiquitin-protein ligase HERC1 | 22 | 52 | 0,33872 | 1 |
| [Q56UN5](http://www.uniprot.org/uniprot/Q56UN5) | M3K19_HUMAN | Mitogen-activated protein kinase kinase kinase 19 | 33 | 11 | 0,32766 | 1 |
| [O60522](http://www.uniprot.org/uniprot/O60522) | TDRD6_HUMAN | Tudor domain-containing protein 6 | 16 | 14 | 0,31967 | 1 |
| [Q03181](http://www.uniprot.org/uniprot/Q03181) | PPARD_HUMAN | Peroxisome proliferator-activated receptor delta | 18 | 7 | 0,31483 | 1 |
| [Q99590](http://www.uniprot.org/uniprot/Q99590) | SCAFB_HUMAN | Protein SCAF11 | 16 | 19 | 0,30675 | 1 |
| [P37059](http://www.uniprot.org/uniprot/P37059) | DHB2_HUMAN | Estradiol 17-beta-dehydrogenase 2 | 26 | 6 | 0,30202 | 1 |
| [P82987](http://www.uniprot.org/uniprot/P82987) | ATL3_HUMAN | ADAMTS-like protein 3 | 17 | 6 | 0,28241 | 1 |
| [Q9H3K2](http://www.uniprot.org/uniprot/Q9H3K2) | GHITM_HUMAN | Growth hormone-inducible transmembrane protein | 34 | 10 | 0,27785 | 1 |
| [Q8WZ42](http://www.uniprot.org/uniprot/Q8WZ42) | TITIN_HUMAN | Titin | 16 | 253 | 0,27782 | 1 |
| [Q14204](http://www.uniprot.org/uniprot/Q14204) | DYHC1_HUMAN | Cytoplasmic dynein 1 heavy chain 1 | 29 | 37 | 0,27623 | 1 |
| [Q9HCU4](http://www.uniprot.org/uniprot/Q9HCU4) | CELR2_HUMAN | Cadherin EGF LAG seven-pass G-type receptor 2 | 25 | 12 | 0,26167 | 1 |
| [Q12955](http://www.uniprot.org/uniprot/Q12955) | ANK3_HUMAN | Ankyrin-3 | 19 | 35 | 0,26077 | 1 |
| [Q9P219](http://www.uniprot.org/uniprot/Q9P219) | DAPLE_HUMAN | Protein Daple | 22 | 33 | 0,25607 | 1 |
| [P98164](http://www.uniprot.org/uniprot/P98164) | LRP2_HUMAN | Low-density lipoprotein receptor-related protein 2 | 17 | 52 | 0,24812 | 1 |
| [O75781](http://www.uniprot.org/uniprot/O75781) | PALM_HUMAN | Paralemmin-1 | 32 | 12 | 0,24692 | 1 |
| [Q9Y2I7](http://www.uniprot.org/uniprot/Q9Y2I7) | FYV1_HUMAN | 1-phosphatidylinositol 3-phosphate 5-kinase | 26 | 12 | 0,24339 | 1 |
| 2A5A_HUMAN | 2A5A_HUMAN | Serine/threonine-protein phosphatase 2A 56 kDa regulatory subunit alpha isoform | 20 | 10 | 0,24077 | 1 |
| [Q9NQC3](http://www.uniprot.org/uniprot/Q9NQC3) | RTN4_HUMAN | Reticulon-4 | 34 | 9 | 0,23625 | 1 |
| [Q14781](http://www.uniprot.org/uniprot/Q14781) | CBX2_HUMAN | Chromobox protein homolog 2 | 31 | 18 | 0,23011 | 1 |
| [O75330](http://www.uniprot.org/uniprot/O75330) | HMMR_HUMAN | Hyaluronan mediated motility receptor | 15 | 17 | 0,21973 | 1 |
| [Q99700](http://www.uniprot.org/uniprot/Q99700) | ATX2_HUMAN | Ataxin-2 | 17 | 11 | 0,20649 | 1 |
| [O75962](http://www.uniprot.org/uniprot/O75962) | TRIO_HUMAN | Triple functional domain protein | 17 | 21 | 0,20507 | 1 |
| [Q6ZU80](http://www.uniprot.org/uniprot/Q6ZU80) | CE128_HUMAN | Centrosomal protein of 128 kDa | 22 | 20 | 0,20185 | 1 |
| [Q6NUI6](http://www.uniprot.org/uniprot/Q6NUI6) | CHADL_HUMAN | Chondroadherin-like protein | 18 | 6 | 0,17849 | 2 |
| [Q9Y277](http://www.uniprot.org/uniprot/Q9Y277) | VDAC3_HUMAN | Voltage-dependent anion-selective channel protein 3 | 129 | 9 | 0,17523 | 3 |
| [Q86YW9](http://www.uniprot.org/uniprot/Q86YW9) | MD12L_HUMAN | Mediator of RNA polymerase II transcription subunit 12-like protein | 23 | 8 | 0,15462 | 1 |
| [Q56UN5](http://www.uniprot.org/uniprot/Q56UN5) | M3K19_HUMAN | Mitogen-activated protein kinase kinase kinase 19 | 16 | 22 | 0,15268 | 2 |
| [A6NME0](http://www.uniprot.org/uniprot/A6NME0) | SPDL1_HUMAN | Putative speedy protein-like protein LOC442572 | 28 | 10 | 0,15177 | 1 |
| [P36776](http://www.uniprot.org/uniprot/P36776) | LONM_HUMAN | Lon protease homolog, mitochondrial | 17 | 5 | 0,1499 | 1 |
| [O75916](http://www.uniprot.org/uniprot/O75916) | RGS9_HUMAN | Regulator of G-protein signaling 9 | 40 | 13 | 0,14975 | 1 |
| [Q6ZN55](http://www.uniprot.org/uniprot/Q6ZN55) | ZN574_HUMAN | Zinc finger protein 574 | 21 | 8 | 0,12939 | 1 |
| [Q9NZJ4](http://www.uniprot.org/uniprot/Q9NZJ4) | SACS_HUMAN | Sacsin | 19 | 51 | 0,11654 | 3 |
| [Q6UB99](http://www.uniprot.org/uniprot/Q6UB99) | ANR11_HUMAN | Ankyrin repeat domain-containing protein 11 | 22 | 51 | 0,11363 | 1 |
| [Q86W56](http://www.uniprot.org/uniprot/Q86W56) | PARG_HUMAN | Poly(ADP-ribose) glycohydrolase | 14 | 12 | 0,09265 | 1 |
| [Q5TF21](http://www.uniprot.org/uniprot/Q5TF21) | SOGA3_HUMAN | Protein SOGA3 | 19 | 16 | 0,06423 | 1 |
| [Q9NXG0](http://www.uniprot.org/uniprot/Q9NXG0) | CNTLN_HUMAN | Centlein | 21 | 38 | 0,05173 | 1 |
| [Q8IVW4](http://www.uniprot.org/uniprot/Q8IVW4) | CDKL3_HUMAN | Cyclin-dependent kinase-like 3 | 16 | 7 | 0,02725 | 1 |
| [Q13459](http://www.uniprot.org/uniprot/Q13459) | MYO9B_HUMAN | Unconventional myosin-Ixb | 15 | 27 | 0,01595 | 1 |
| [P19021](http://www.uniprot.org/uniprot/P19021) | AMD_HUMAN | Peptidyl-glycine alpha-amidating monooxygenase | 65 | 13 | 0,00323 | 1 |
